# Labour Q1 pain – poorly analysed and reported: a systematic review

**DOI:** 10.1186/s12884-018-2089-2

**Published:** 2018-12-07

**Authors:** Hans Järnbert-Pettersson, Linda Vixner

**Affiliations:** 1 Karolinska Institutet - Department of Clinical Science and Education, Södersjukhuset, Stockholm, Sweden; 2 Dalarna University- School of Education, Health and Social Studies, Falun, Sweden

**Keywords:** Labour pain, Repeated-measures data, Longitudinal study, Mixed models, Mixed effect models, Repeated measure ANOVA, Statistical analysis, CONSORT, STROBE

## Abstract

**Background:**

Modelling and analysing repeated measures data, such as women’s experiences of pain during labour, is a complex topic. Traditional end-point analyses such as t-tests, ANOVA, or repeated measures [rANOVA] have known disadvantages. Modern and more sophisticated statistical methods such as mixed effect models provide flexibility and are more likely to draw correct conclusions from data. The aim of this study is to study how labour pain is analysed in repeated measures design studies, and to increase awareness of when and why modern statistical methods are suitable with the aim of encouraging their use in preference of traditional methods.

**Methods:**

Six databases were searched with the English language as a restriction. Study eligibility criteria included: Original studies published between 1999 and 2016, studying pregnant women in labour with the aim to compare at least two methods for labour pain management, with at least two measurements of labour pain separated by time, and where labour pain was analysed.

After deduplication, all records (*n* = 2800) were screened by one of the authors who excluded ineligible publication types, leaving 737 records remaining for full-text screening. A sample of 309 studies was then randomly selected and screened by both authors.

**Results:**

Among the 133 (of 309) studies that fulfilled the study eligibility criteria, 7% used mixed effect models, 20% rANOVA, and 73% used end-point analysis to draw conclusions regarding treatment effects for labour pain between groups. The most commonly used end-point analyses to compare groups regarding labour pain were t-tests (57, 43%) and ANOVA (41, 31%). We present a checklist for clinicians to clarify when mixed effect models should be considered as the preferred choice for analysis, in particular when labour pain is measured.

**Conclusions:**

Studies that aim to compare methods for labour pain management often use inappropriate statistical methods, and inaccurately report how the statistical analyses were carried out. The statistical methods used in analyses are often based on assumptions that are not fulfilled or described. We recommend that authors, reviewers, and editors pay greater attention to the analysis when designing and publishing studies evaluating methods for pain relief during labour.

**Electronic supplementary material:**

The online version of this article (10.1186/s12884-018-2089-2) contains supplementary material, which is available to authorized users.

## Background

Women’s experiences of pain during labour are highly variable [1] and are related to women’s overall assessments of childbirth [2]. A painful labour may have long-term consequences for women’s health and wellbeing [3]. Most women in labour require pain relief, which can be administered in either pharmacological or non-pharmacological form [4].

Comparing labour pain between different treatment groups needs consideration, both regarding when and how often pain assessments are made, and how statistical analyses have been conducted to reflect the reality of women’s pain varying during labour. Women’s pain varies both between individual women and within the same woman over time as well as between different treatment groups. The mean intensity of labour pain is closely related to the progression of labour and increases with greater cervical dilatation [5–10]. In addition, labour pain correlates with the intensity, duration, and frequency of uterine contractions [1, 8]. Furthermore, although pain increases with time, individual progression can differ from woman to woman as a result of both biological factors and the interventions that are administered.

When pain assessments are made more than once for each woman, conclusions made on the management of labour pain will be based on repeated measures data. The most common research questions (and corresponding statistical test) for these study designs are; *1) Is there any difference in mean pain between the treatment group and the control group(s) over all time-points?* (overall treatment effect between groups), 2) *If so, are the differences in labour pain between groups equal or do they differ at different time-points?* (interaction between treatment and time), and 3) *is there a difference in pain within each group?* (within group effect). In these cases, appropriate statistical methods for repeated measures data should be used (rANOVA or mixed models). This is in contrast to analyses in studies where only one time-point is of interest. In such cases, end-point analyses such as t-tests or ANOVA could be used.

The most commonly used methods to measure labour pain are verbal reports with standardised instruments such as the Visual Analogue Scale (VAS), the McGill Pain Questionnaire (MPQ), the Short-Form MPQ, verbal rating scales, or simple ordinal scales [1]. Although there are many studies that consider how labour pain should be assessed [1] in trials evaluating methods for pain relief during labour, to our knowledge there are no studies that have studied how these assessments are analysed and reported.

The purpose of this paper is to study how labour pain is analysed in studies with a repeated measures design, and to increase awareness of when and why modern statistical methods are suitable, so as to encourage their use over traditional methods. This paper will briefly review the most frequently occurring methods of repeated measures analysis for continuous outcomes when means are compared. A summary of aspects to consider when studies with an emphasis on labour pain are planned and analysed and the potential consequences of the choice of statistical method are presented in Table 1, (modified from [11, 12]).

**Table 1:** Comparison of traditional and mixed-effect approaches and questions to answer when choosing a statistical method for the analysis of labour pain in repeated measures data

Questions to ask when you choose statistical method	Statistical property	Statistical method		
		End-point Analysis	rANOVA	Mixed effect models
1. What do you want to compare?	Research question	Compares mean labour pain between groups at one time-point	Compares mean labour pain between groups at several time-points Study interactions between time^a^treatments	a. Compares mean labour pain between groups at several time-points b. Study interactions between time and treatment c. Study individual women’s pain changes over time
2. Do you have measurements of labour pain at all time-points for all women?	Missing data	Excludes woman with missing measurements	Excludes woman with missing measurements	Use all available measurements under the assumption of missing at random (MAR)
	Possible effect of omitting women with missing values?	Sample bias	Sample bias	Not applicable
	Possible effect of imputation of missing data?	Estimation bias	Estimation bias	Not applicable
3. Can you assume that correlation of pain is equal between all time-points?	Assumption on the between woman pain correlation	Independent	Independent	Independent
Are the labour pain assessments made with unequal distances, e.g. at baseline, and after 2 and 6 h?	Assumption on the individual woman pain correlation or covariance matrix	Independent	Compound symmetry	Allow a variety of covariance structures, e.g. Independence, Compound symmetry, AR [1], Unstructured
4. Can you assume that the variance of labour pain is equal at all time-points?	Assumption on the variance of pain at different time-points	Constrained to be equal at all time-points	Constrained to be equal at all time-points	Allowed to vary
5. Are measurements of labour pain normally distributed?	Assumption of normal distribution	Normality assumption	Normality assumption	Normality assumption
6. What requirements do you have on your statistical model to model the pain over time?	Description of time effect	Simple	Flexible	Flexible
7. Would you like to consider labour pain traits for individual women over time?	Estimation of individual trends	No	No	Yes
8. Do you need to adjust labour pain for factors that vary during labour, e.g. cervical dilatation and use of other pain relief?	Time dependent covariates	No	Yes	Yes
9. Do you have knowledge of applied statistics?	Ease of implementation	Very easy	Easy	Complex
10. Do you have access to a good computer?	Computational complexity	Low	Low	High

### Traditional methods

End-point analysis compares mean pain between treatments and control groups at one specific time-point, or a difference between two specific time-points (e.g. baseline versus last measurement). Independent t-tests assume that two groups are compared while ANOVA or ANCOVA are used if three or more groups are compared, and these are often followed by independent t-tests to study pairwise comparisons. This approach tests the mean difference between groups at a specific time-point rather than testing whether there is a general difference between the groups over all relevant time-points. Although easy to understand and implement, it is well known that the probability of a type I error (false positive) increases when using this approach if comparisons are done at each time-point, which implies that the conclusion might falsely be that a treatment has an effect on labour pain). Furthermore, it is not possible to formally test if there are any interactions between time and treatment since only one time-point is considered at a time.

In contrast to end-point analysis, repeated measures ANOVA (rANOVA) includes data from all time-points in the analysis. It makes it possible for the total increase in mean pain to be equal between baseline and the final measurement in both groups, even though there is a difference in mean pain score among the groups at earlier time-points. In such cases, an end-point analysis will find differences at the final time point but not at earlier time-points. Thus, end-point analysis cannot, in contrast to rANOVA, give any answer to if there is an overall difference in mean pain between the groups with respect to all time points, which is often the research question. In addition, rANOVA can formally find if the differences are similar at all time-points by using the statistical test for interactions between treatment and time.

There are, however, disadvantages in using rANOVA in the context of analysing labour pain. The assumptions for this model to make appropriate conclusions regarding treatment effects are rarely met in practice. Firstly, rANOVA requires the same number of repeated measurements to be made for all women. Hence, even one missing pain measurement at one time point for one woman, would lead to all data collected from this woman to be lost, i.e. each woman with missing data will be excluded from the analyses. There are two ways to circumvent this missing data problem when using rANOVA; either data need to be imputed, which in turn might lead to an estimation bias, or analyses could be conducted only on data from the group of women with complete data sets, which might introduce a sample bias since these women may not be representative for the whole population. In addition, statistical power will be reduced.

Secondly, an assumption that is implicit for rANOVA is the sphericity assumption, which implies that variance is assumed to be equal at all times (constant variance) and that the covariance (correlation) between any two time-points is equal. These assumptions are usually not met when labour pain is analysed since consecutive pain measurements on the same woman tend to be more correlated than measurements taken further away in time. For example, the correlation between a woman’s pain assessments at the two first measurements (which are often measured with a short time interval between them) are often higher than the correlation between the first and last pain measurement. When rANOVA is used despite not fulfilling the assumptions, there is a risk for type I errors when the interaction between treatment and time is tested, which in turn might lead to incorrect conclusions about the question of whether there is a difference in labour pain at different time-points .

### Modern methods

Using mixed effect models is a more flexible method of analysing labour pain than traditional methods as they allow correlated data, variances that differ between time-points and have greater flexibility in handling missing data [13, 14]. They have a number of advantages, the first being that mixed effect models can account for the correlation between measurements, which implies that mixed effect models can handle uneven time intervals between measurements, such as when pain is measured more frequently during the first hour after an intervention than in the later stages of labour (e.g. at 15, 30, 45, 60, 90, 150 min). These correlations can be modelled, which implies that, for example, the assumption of data sphericity in rANOVA does not need to be met. In addition, the variance is also allowed to vary between time-points that enable the model to allow for greater variance in labour pain at time-points closer to birth.

The second advantage is that mixed effect models can use all available measurements for all women and therefore handle missing data more appropriately – as long as they meet the missing-completely-at-random definition [15]. Data are missing completely at random (MCAR) if the occurrence of missing data is independent of both observed and unobserved outcomes. If a woman drops out of a study due to treatment-related adverse effects, any lacking measurements of pain for this patient is classified as missing-at-random (MAR). Mixed effect models can take care of missing data as long as they meet the assumptions of MCAR. In contrast, in these cases traditional methods require the exclusion of all data from a woman with partially missing data in the analysis.

The third advantage is that mixed models can manage between-individual heterogeneity and that pain for each individual woman will deviate randomly from the overall average pain (random intercept), and that the treatment effect also will differ among the women (random treatment effect). This is in contrast to traditional models, which assume that average pain is equal for all women at baseline (i.e. no random intercept) and that the treatment effects are equal for all women (i.e. no random effect of treatment) in all groups.

## Methods

### Study selection

We followed the preferred reporting standard for systematic reviews (PRISMA) [16] to review studies with a repeated measures design and with labour pain analysed as a continuous outcome. Our searches were limited by date (published between Jan 1, 1999, and March 7, 2016) and included the following databases: Medline (OVID), Cinahl (Ebsco), Cochrane (Wileys), Embase (Embase.com), PubMed (complement), and WoS (Thomson Reuters). The search was conducted by librarians at Karolinska Institutet’s University Library. Search terms used for each database can be found in Additional file 1: Appendix S1. Inclusion criteria were: original studies, studying pregnant women in labour intending a vaginal birth (any parity or risk status), with the aim to compare at least two methods for labour pain management, with at least two measurements of labour pain at different time points, and where labour pain was analysed or presented as a mean.

**Fig. 1:**
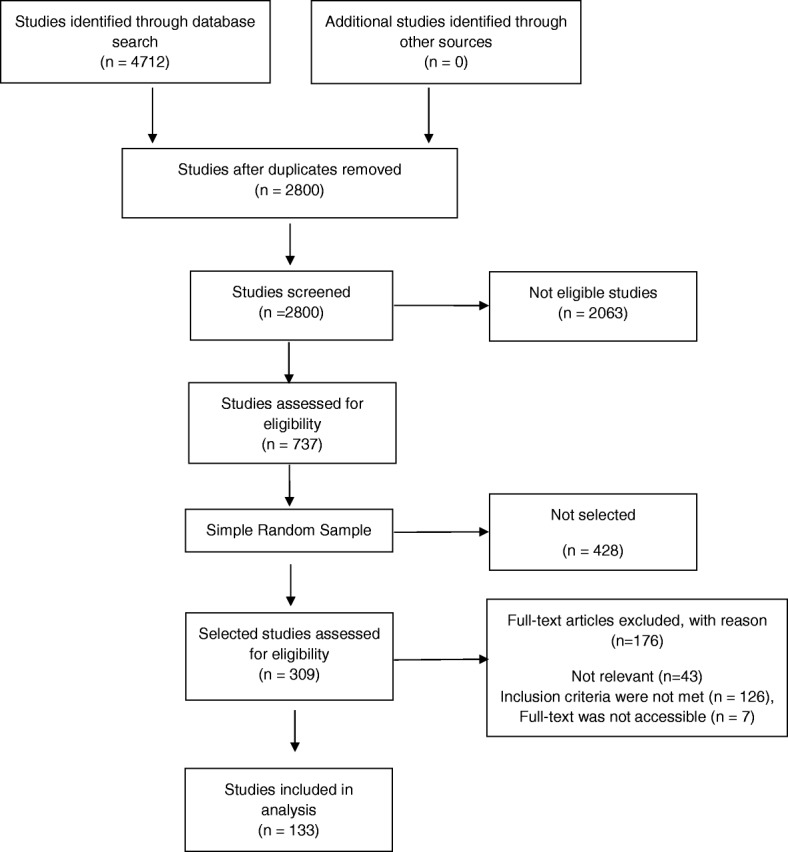
Flow chart for inclusion of studies

The total number of studies after deduplication was 2800, which were all screened by one of the authors to exclude ineligible publication types (including, letters, editorials, and reviews), animal or in vitro research, and studies published in a language other than English (Fig. 1). After the screening process, 737 studies remained eligible for full-text screening.

A sample size calculation were performed to assure that we could estimate the proportion of studies that used mixed models with a high precision (95% confidence interval with a precision of plus-minus 4 percentage points). We assumed that the proportion of studies that used mixed effect models, would be 5%. The sample size calculation gave 150 studies; in addition, we assumed that approximately 50% would fulfil our inclusion and exclusion criteria. A sample of 309 studies were therefore randomly selected from the 737 studies assessed eligible for full-text screening.

### Data collection process

Of the 309 selected studies that were read in full-text, 175 did not fulfil the inclusion criteria, leaving 133 that were included (Fig. 1) from 54 different journals (Additional file 2: Table S1). We developed a data extraction sheet, Additional file 3: Appendix S2, with items modified from the CONSORT [17] and STROBE [18] checklists. In addition, information on study design, instruments for pain assessment including anchor labels, time-points, and intervals for pain assessments, number of comparison groups, effect measure for labour pain, number of observations in the analysis, tests for normality, and management of missing data were recorded in the data extraction sheet. We also examined if there were any discrepancies between the statistical method section and the reporting in the result section.

Both authors pilot-tested the sheet on 30 randomly selected included studies. Thereafter, both authors independently read all the studies and assessed the most advanced statistical method used in the following order: mixed-effect model, rANOVA, ANOVA, and end-point analysis. Disagreements between the authors’ assessments were resolved by reading the studies again followed by a discussion between the two review authors.

### Statistical methods

We present data from our sample as frequencies and percentages that represent an estimate of the occurrence in the population from which the sample of studies intends to generalize the results to. To illustrate the precision in the estimates (highest uncertainty around 50% and lowest close to 0%), we generated binomial exact confidence intervals (Clopper Pearson) for the following percentages: 1, 5, 10, 20, 50, and 90% presented in Tables 2 and 3. Analysis was conducted using SPSS version 23, and the pre-specified sample size calculation was conducted using Sample Power 3.0.

**Table 2:** Repeated measures designs in 133 labour pain studies

		Number	Percent (%)
Study Design	Experiment - randomised	116	87.2
	Experiment - not randomised	13	9.8
	Observational - Cohort	3	2.3
	Not clear	1	0.8
Number of groups compared	2	96	72.2
	3	24	18.0
	> 4	13	9.8
Total number of women included in analysis	20–30	10	7.5
	31–50	32	24.1
	51–100	55	41.4
	101–500	32	24.1
Pain - number of time points measured	2	14	10.5
	3–5	48	36.1
	6–10	56	42.1
	> 10	15	11.3
Equally spaced time intervals	No	92	69.2
	Yes	40	30.1
	Not clear	1	0.8
Pain - outcome measure	Primary outcome	11	8.3
	Secondary outcome	4	3.0
	Not explicit	118	88.7
Pain - measurement Instrument^a^	VAS (Visual analogue scale)	110	82.7
	NRS (numeric rating scale)	6	4.5
	MPQ, short version	1	0.8
	Verbal rating scale	14	10.5
	Other	2	1.5

^a^Anchor labels for the instruments were specified in 65% (67/133)

Illustrative binomial exact 95% confidence intervals for percentages when sample size is 133: 1% (0 to 4%); 5% (2 to 10%); 10%(5 to 16%); 25%(18 to 33%); 50%(40 to 58%); 90(84 to 95%)

**Table 3:** Statistical methods and presentation of results in 133 labour pain studies with repeated measures design

		Number	Percent (%)
Most advanced statistical method to analyse labour pain	End-point analysis^a^	97	72.9
	rANOVA	26	19.5
	Mixed effect models	9	6.8
	Not clear	1	0.8
Clear how between-groups comparisons are conducted?	No	34	25.6
	Yes	99	74.4
Are comparison conducted within groups (between time points)?	No	87	65.4
	Yes	46	34.6
Clear how within group comparisons are conducted?	No	19	41.3
	Yes	27	58.7
Are numbers of valid observations used in analysis for each group and time-point stated?	No - only number of individuals at one time-point, e.g. baseline is stated	112	84.2
	Yes - number of individuals/valid measurements at each time-point is stated	21	15.8
Is normality assumption tested/mentioned?	No	120	90.2
	Yes	13	9.8
Clear agreement between statistical methods and results presented?	No	77	57.9
	Yes	56	42.1

^a^Several end-point analyses could be used in each of these 97 studies: t-tests were used in 59 (44%) studies, ANOVA in 41 studies (31%), Mann-Whitney in 20 studies (15%), Wilcoxon in 6 studies (5%), and ANCOVA in 1 study (1%)

Illustrative binomial exact 95% confidence intervals for percentages when sample size is 133: 1% (0 to 4%); 5% (2 to 10%); 10%(5 to 16%); 25%(18 to 33%); 50%(40 to 58%); 90(84 to 95%)

## Results

The most commonly used study design was a randomised controlled trial (87%) with two or three experiment groups (Table 2). Analyses of labour pain were conducted including > 51 women in 66% of the studies. Measurements of labour pain were made repeatedly for each woman between 3 and 10 times in 90% of the studies, and the time intervals between each measurement was equal in 31% of the studies. Labour pain was rarely explicitly defined as a primary or secondary outcome.

A Visual Analog Scale (VAS) [19] was the most-often used tool to measure labour pain (used in 110 studies [83%]), although the description of the scale differed among the studies, or the scale was not clearly described. The maximum anchor labels of VAS were in 60% (66/110) of the studies. Among these 66 studies, we found 23 different definitions. The most commonly used anchor labels for maximum pain were “worst pain imaginable” (*n* = 17), “worst possible pain” (*n* = 10), “worst pain” (*n* = 10), “worst imaginable pain” (*n* = 9), “unbearable pain” (*n* = 4), and “severe pain” (*n* = 3). For the minimum anchor label of VAS, we found four definitions among the 67 studies where it was defined, with the most common definitions being “no pain” (*n* = 60) and “no pain at all” (*n* = 4).

Statistical methods used to compare labour pain between treatment groups are presented in Table 3. Among the 133 included studies, Mixed effect models were the most advanced statistical method found in 9 studies (7%), rANOVA in 26 studies (20%), and end-points analysis in 97 studies (73%). The most frequently used statistical methods to analyse repeated measures data were t-tests and ANOVA.

Among the 123 studies that used end-point or rANOVA to analyse labour pain, the specific number of observations used in the analysis at each time-point were stated in only 16% of the studies. In 85 of the 123 studies (69%), pain was assessed at time-points with unequal distances between them. It was not clear whether there were missing data, and/or if the measurements of labour pain were made at time-points with unequal distances in 108 of the 123 studies (88%), which used end-point analysis or rANOVA.

There was no clear presentation of how between-group comparisons were made in 34 of the 133 studies (26%). In the 46 of the 133 studies (35%) where within-group comparisons were made (i.e. comparison within a group between time-points), it was not clear how these comparisons had been made in 19 (41%) of them.

There was clear agreement between the statistical methods presented in the method section of the paper and the result section in only 42% of the studies. For example, the method section might state that ANOVA was used to compare labour pain between the groups; however *p*-values in the results section did not specify at which time points comparisons were made, or whether the p-value corresponded to an overall test between three groups made by an ANOVA or by a post-hoc t-test between two specific groups. How, or if, assumptions regarding normality were examined was reported in only 10% of the studies.

## Discussion

This systematic review found that studies aiming to compare at least two methods of labour pain management often use inappropriate statistical methodology, and inaccurately report how the statistical analyses were conducted. In particular:

1. Statistical models used in the analyses were more often than not based on incorrect assumptions to give an appropriate answer to the research question. End-point analysis or ANOVA were used to test for differences between groups over time, though these methods only formally test for differences at a specific time-point.
2. How the analyses were conducted was often not clearly described. This means that the reader has to make assumptions about how the analyses have been carried out. In particular, missing data at different time-points were rarely reported.
3. The verbal anchor descriptors of the end-points of VAS, time intervals between each pain assessment, and the number of pain assessments varied largely in the studies.

Problems associated with the poor application and reporting of statistical methods are not new. Altman (1982) [20] noted decades ago that researchers both use the wrong techniques (wilfully or in ignorance) and use the right techniques incorrectly. Our study supports this finding, as the quality of the studies included here makes it difficult for readers to assess the value of the conclusions regarding labour pain management.

The use of more advanced statistical methods has increased in recent decades, and continues to do so [21–23]. In general, there is a natural time lag between new statistical methods being developed and being used and applied in research. Mixed models were developed during the 1980s and implemented in statistical software programs such as SAS, STATA, SPSS and R in the following decades. In contrast to other research areas where there has been a debate on methodological aspects regarding how to analyse data that is measured repeatedly with mixed models [11], we have found no such debate in the area of labour pain research.

Thus, the prerequisites for using mixed models in the context of measuring labour pain in this study show that analyses are rarely carried out using an approach that reflects how data are collected. If and how the conclusions regarding the management of labour pain would change if appropriate analyses were conducted remains unknown.

Statements such as CONSORT [17] and STROBE [18] give recommendations on how statistical methods and results should be reported with the principle to follow; *“Describe statistical methods with enough detail to enable a knowledgeable reader with access to the original data to verify the reported results (**www.icmje.org**)*” [17]. In this study we did not only assess whether the statistical methods used were clearly described in the methods section (CONSORT, item 12a), but we also assessed if there was a clear agreement between the methods section and the results section, and whether the choice of statistical methods was appropriate for answering the research question in the context of repeated measured data. Our results should be interpreted with this in mind, and for clarity we would like to emphasise the following points. A clear description of the statistical methods used in the methods section does not necessarily mean that those methods were actually used, or that these methods were used appropriately. Also, a clear description of the method and clear agreement in the results section does not necessarily mean that the used method was appropriate to answer the research question.

We noted a few typical reporting errors, for example that the statistical method section described several methods that had been used, however, as a reader, when reading the results section you needed to make assumptions as to which test or model had been used. The method section could describe both t-tests and Mann-Whitney U-tests, but in the results section there was no information as to which of the methods had been used for specific results. Other examples such as the use of ANOVA without a description of which model was used (e.g. with/without interactions, adjusted/not adjusted for confounders), commonly occurred. We also found studies where the method section seemed to be completely independent from the results section, as if the method section appertained to another study, and was not in accordance with the study’s research question at all. In addition, we found studies that we suspected contained simple misspellings or typos, for example, a methods section could describe that a dependent t-test was used to compare differences between groups, which raises the question whether this was the case, or if actually it was an ***in***dependent t-test that had been used.

The problem of using inappropriate statistical methods even though the statistical methods were clearly described was also quite common. For example, the methods section could describe that a t-test was used, which was also in clear agreement with the results section (which is often the case if a single method is used), but this occurred in cases where the method was not appropriate. One might argue that a “simple test” need not be presented in detail, which motivates the use of sentences such as “t-tests, rANOVA and Mann Whitney U tests were used as appropriate”. The problem is that what “appropriate use” is according to professional statisticians and applied researchers seems to differ somewhat.

Although most of the included studies were randomised and should follow the CONSORT statement for reporting, the recommendation to describe statistical methods in detail was not followed. One explanation for this lack of detail could be that not all journals have yet endorsed CONSORT, or had not endorsed CONSORT at the time of the publication of the included study. It has been shown that journal endorsement of CONSORT may benefit the completeness of the reporting of randomised trials [24].

A strength of this study is that generalizations can be made to all studies that compare the management of labour pain between groups over time with a continuous outcome, i.e. studies with a repeated measures design. In contrast to studies that investigate methodological aspects of analysis and design, we did not include studies from specific journals [21–23, 25] or conduct our search strategy with keywords only regarding study design. Only 13% (17/133) of our included studies had the keywords “longitudinal” or “repeated measures”. Instead, we conducted our search with a subject of medical research interest, i.e. pain among women in labour, and then manually classified the studies with regard to study design, which implies that this systematic review reflects how studies in general analyse and report labour pain, not how this is done in specific journals or in studies with a specified study design based on search words such as “longitudinal” which we believe are more aware of the analysis of repeated measures [12].

Women’s perceptions of labour pain intensity vary greatly during labour, and the pattern of pain differs between women for a number of reasons, e.g. it differs between nulliparous and multiparous women and between women in early and later stages of labour [1]. Most methods for labour pain relief are safe for both mother and baby, however, their efficacy is unclear due to limited methodological quality. The way pain has been assessed differs considerably among the studies and it is not always clear which tool was used to assess pain or how the extremes of the scales were marked (anchor labels). In addition, information about pain relief at different stages of labour are also lacking [4]. The findings in our study confirm that end-points on the pain assessment scales differ considerably, which has been confirmed elsewhere [26]. High-quality systematic reviews and other synthesized research evidence compiled by HTA (Health Technology Assessment) organisations around the world, is the base for clinicians to make informed health decisions. As noted by others, to assess and analyse labour pain without considering the aspects of variability during labour, limitations in more traditional statistical methods, and without considering the fact that there are no specific recommendations with respect to anchor labels makes compiling evidence in e.g. systematic reviews challenging [4].

To improve design, analysis and reporting we recommend that authors, reviewers, and editors:

1. In addition to e.g. CONSORT and STROBE checklists, use extraction sheet Additional file 3: Appendix S2, the questions 7–22 to check if your article answer these questions.
2. Use Table 1 to choose statistical method and to check if the analysis is appropriate reported with respect to your research question and design. Consult a professional statistician if you are insecure about the answers and which statistical method that is appropriate and why.
3. Don’t forget point 1 and 2. You should be aware of the questions (and answers) already in the design of your study. To press buttons in a statistical software program is not a valid foundation for the results if you are not aware about the assumptions underlying the results. The statistical analysis is the foundation for your results and, in turn, your conclusions about treatments of labour pain – don’t forget that.

## Conclusions

Studies aiming to compare at least two methods for labour pain management often use inappropriate statistical methods, and inaccurately report how statistical analyses were carried out. The verbal anchor descriptors of pain assessments, the time intervals between each pain assessment, and the number of pain assessments made varies largely among the studies. The statistical methods used in the analyses are often based on assumptions that are either not fulfilled or not described, or both. We recommend that authors, reviewers, and editors pay greater attention to method choice and use when designing, reviewing, and publishing studies evaluating methods for pain relief during labour.

## Additional files

Additional file 1: **Appendix S1.** Search strategy for the six databases. (DOCX 19 kb)

Additional file 2: **Table S1.** Included Journals. (DOCX 14 kb)

Additional file 3: **Appendix S2.** Data extraction sheet used to collect data. (DOCX 32 kb)

## Abbreviations

ANCOVA: Analysis of covariance
ANOVA: Analysis of variance
AR: Auto Regressive
CONSORT: Consolidated standards of reporting trials
MAR: Missing at random
MCAR: Missing completely at random
MPQ: McGill Pain Questionnaire
NRS: Numeric rating scale
PRISMA: Preferred reporting standard for systematic review
rANOVA: Repeated measure ANOVA
STROBE: Strengthening the Reporting of observational studies in Epidemiology
VAS: Visual analogue scale
