# Supplementary material for: Labour Q1 pain – poorly analysed and reported: a systematic review
Source: BMC Pregnancy Childbirth. 2018 Dec 7;18:483. doi: 10.1186/s12884-018-2089-2 (PMC6286546; doi:10.1186/s12884-018-2089-2)
Supplement: Supplementary file 1 — Appendix S1. Search strategy for the six databases. (DOCX 19 kb) [file 12884_2018_2089_MOESM1_ESM.docx]

**Additional file 1: Appendix S1** Search strategy for the six databases.

**1. Medline (OVID)**

| Date of Search: 2016.03.07  Number of hits: 1067  Comments: | Field tags:   - .tw. = title & abstract - exp/ = MeSH, exploded - kf = keywords |
| --- | --- |
| 1. Labor Pain/  2. Labor, Obstetric/  3. exp Parturition/  4. Delivery, Obstetric/  5. Labor, Induced/  6. Obstetric Labor Complications/  7. ((Labor or labour) adj3 pain).tw,kf.  8. (labor or labour or childbirth or parturition or ((obstetric* or vagin*) adj3 delivery)).tw,kf.  9. or/1-8  10. Analgesia, Obstetrical/  11. Analgesia, Epidural/  12. Anesthetics, Local/  13. Analgesics, Opioid/  14. Nerve Block/  15. Acupuncture Analgesia/  16. exp Acupuncture Therapy/  17. Acupressure/  18. Baths/  19. Breathing Exercise/  20. Electroacupuncture/  21. Homeopathy/  22. Hydrotherapy/  23. Hypnosis/  24. Immersion/  25. Massage/ 26. Phytotherapy/  27. Pain Management/  28. Posture/  29. exp Plant Preparations/  30. Relaxation Therapy/  31. Transcutaneous Electric Nerve Stimulation/  32. (analgesi* or anesthet* or acupress* or acupuncture or nerve block or bath* or breathing or electroacupuncture or homeopathy or hydrotherapy or hypnosis or immersion or massage or phytotherapy or relaxation or TENS).tw,kf.  33. ((electric or nerve) adj3 stimulation).tw,kf.  34. (sterile adj3 water).tw,kf.  35. (pain adj3 (management or prevent* or relief or treat*)).tw,kf.  36. non-pharmacologic*.tw,kf.  37. or/10-36  38. 9 and 37  39. Pain Measurement/  40. Visual Analog Scale/  41. Pain assessment.tw,kf.  42. Verbal rating scale.tw,kf.  43. Numeric rating scale.tw,kf.  44. ((rat* or measurement or scale* or score* or screen* or questionnaire* or test* or assessment* or VAS or NRS or VRS) adj5 pain).tw,kf.  45. Double-Blind Method/  46. double-blind*.tw,kf.  47. Treatment Outcome/  48. outcome assessment.tw,kf. 49. or/39-48  50. 38 and 49  51. limit 50 to (english language and yr="1999 -Current")  52. exp animals/ not humans.sh.  53. 51 not 52  54. remove duplicates from 53 | |

**2. Cinahl (Ebsco)**

| Date of Search: 2016.03.07  Number of hits: 600  Comments: | Field tags:   - MH = Cinahl Heading - TI/AB = Title, Abstract |
| --- | --- |
| S1 (MH "Labor Pain")  S2 (MH "Labor")  S3 (MH "Delivery, Obstetric")  S4 (MH "Vaginal Birth")  S5 (MH "Childbirth")  S6 (MH "Labor, Induced")  S7 (MH "Labor Complications")  S8 TI ( ((Labor or labour) N3 pain) ) OR AB ( ((Labor or labour) N3 pain) )  S9 TI ( (labor or labour or childbirth or parturition or ((obstetric* or vagin*) N3 delivery)) ) OR AB ( (labor or labour or childbirth or parturition or ((obstetric* or vagin*) N3 delivery)) )  S10 S1 OR S2 OR S3 OR S4 OR S5 OR S6 OR S7 OR S8 OR S9  S11 (MH "Analgesia, Obstetrical")  S12 (MH "Analgesia, Epidural")  S13 (MH "Anesthetics, Local")  S14 (MH "Analgesics, Opioid")  S15 (MH "Nerve Block")  S16 (MH "Acupuncture+")  S17 (MH "Acupressure")  S18 (MH "Bathing and Baths")  S19 (MH "Breathing Exercises")  S20 (MH "Homeopathy")  S21 (MH "Hydrotherapy+")  S22 (MH "Hypnosis")  S23 (MH "Hypnosis, Anesthetic")  S24 (MH "Immersion")  S25 (MH "Massage")  S26 (MH "Naturopathy")   S27 (MH "Posture")  S28 (MH "Transcutaneous Electric Nerve Stimulation")  S29 TI ( (analgesi* or anesthet* or acupress* or acupuncture or nerve block or bath* or breathing or electroacupuncture or homeopathy or hydrotherapy or hypnosis or immersion or massage or phytotherapy or relaxation or TENS ) OR AB ( (analgesi* or anesthet* or acupress* or acupuncture or nerve block or bath* or breathing or electroacupuncture or homeopathy or hydrotherapy or hypnosis or immersion or massage or phytotherapy or relaxation or TENS ) S30 TI ( ((electric or nerve) N3 stimulation) ) OR AB ( ((electric or nerve) N3 stimulation) ) S31 TI (sterile N3 water) OR AB (sterile N3 water)  S32 TI ( (pain N3 (management or prevent* or relief or treat*)) ) OR AB ( (pain N3 (management or prevent* or relief or treat*)) )  S33 TI non-pharmacologic* OR AB non-pharmacologic*  S34 S11 OR S12 OR S13 OR S14 OR S15 OR S16 OR S17 OR S18 OR S19 OR S20 OR S21 OR S22 OR S23 OR S24 OR S25 OR S26 OR S27 OR S28 OR S29 OR S30 OR S31 OR S32 OR S33 S35 S10 AND S34  S36 (MH "Pain Measurement")  S37 (MH "Visual Analog Scaling")  S38 TI "Pain assessment" OR AB "Pain assessment"  S39 TI "Verbal rating scale" OR AB "Verbal rating scale"  S40 TI "Numeric rating scale" OR AB "Numeric rating scale"  S41 TI ( ((rat* or measurement or scale* or score* or screen* or questionnaire* or test* or assessment* or VAS or NRS or VRS) N5 pain) ) OR AB ( ((rat* or measurement or scale* or score* or screen* or questionnaire* or test* or assessment* or VAS or NRS or VRS) N5 pain) )  S42 (MH "Double-Blind Studies")  S43 TI double-blind OR AB double-blind  S44 (MH "Treatment Outcomes")  S45 TI "outcome assessment" OR AB "outcome assessment"  S46 S36 OR S37 OR S38 OR S39 OR S40 OR S41 OR S42 OR S43 OR S44 OR S45  S47 S35 AND S46  Published Date: 19900101- 20161231. Language0: - English | |

**3. Embase (Embase.com)**

| Date of Search: 2016.03.07  Number of hits: 1745  Comments: | Field tags:   - ab,ti = abstract & title - /exp = Emtree, exploded - /de = Emtree, not exploded |
| --- | --- |
| 1. ‘Labor Pain’/de  2. ‘Childbirth’/exp  3. ‘Delivery’/de  4. ‘Labor induction’/de  5. 'Labor management'/de  6. ‘Labor Complication’/de  7. 'Vaginal delivery'/de  8. 'Natural childbirth'/de  9. 'Water birth'/de  10. ((Labor or labour) NEAR/3 pain):ab,ti  11. (labor or labour or childbirth or parturition or ((obstetric* or vagin*) NEAR/3 delivery)):ab,ti  12.. or/1-11  13. ‘Obstetric anesthesia/exp  14. ‘electroanalgesia’/de  15. ‘Epidural anesthesia’/de  16. ‘Local anesthetic agent ‘/de  17. ‘Narcotic analgesic agent’/de  18. ‘Nerve Block’/de  19. ‘Cervical plexus block’/de  20. Acupuncture/exp  21. ‘Bath’/de  22. ‘Breathing Exercise’/de  23. ‘Homeopathy’/de  24. ‘Hydrotherapy’/de  25. ‘Hypnosis’/de  26. ‘Immersion’/de  27. ‘Massage’/de  28. ‘Phytotherapy’/de  29. ‘Body posture’/de  30. ‘Relaxation Training’/de  31. ‘Plant extract’/de  32. ‘Transcutaneous nerve stimulation’/de 33. (analgesi* or anesthet* or acupress* or acupuncture or nerve block or bath* or breathing or electroacupuncture or homeopathy or hydrotherapy or hypnosis or immersion or massage or ((electric or nerve) NEAR/3 stimulation) or phytotherapy or relaxation or TENS or (sterile NEAR/3 water)):ab,ti 34. (pain NEAR/3 (management or prevent* or relief or treat*)):ab,ti 35. (non-pharmacologic*):ab,ti 36. or/13-35 37. 12 and 36 38. ‘Pain Measurement’/de 39. ‘Pain assessment’/exp 40. (“Pain assessment” or “pain measurement”):ab,ti 41. (“Verbal rating scale”):ab,ti 42. (“Numeric rating scale”):ab,ti 43. ((measurement OR rat* OR scale* OR score* OR screen* OR questionnaire* OR test* OR assessment* OR VAS OR NRS OR VRS) NEAR/5 pain):ab,ti 44. ‘Double blind procedure’/de 45. (double-blind*):ab,ti 46. ‘Treatment Outcome’/de 47. 'outcome assessment':ab,ti 48. or/37-47 49. 37 and 48 50. #12 AND #36 AND #49 AND [english]/lim AND [1999-2016]/py | |

**4. Cochrane (Wileys)**

| Date of Search: 2016.03.07  Number of hits: 34 reviews, 681 trials  Comments: | Field tags:   - ti,ab = title & abstract |
| --- | --- |
| #1 ((Labor or labour) near/3 pain):ti,ab #2 (labor or labour or childbirth or parturition or ((obstetric* or vagin*) near/3 delivery)):ti,ab #3 #1 or #2 #4 (analgesi* or anesthet* or acupress* or acupuncture or nerve block or bath* or breathing or electroacupuncture or homeopathy or hydrotherapy or hypnosis or immersion or massage or phytotherapy or relaxation or TENS or non-pharmacologic* or "nerve stimulation" or "sterile water"):ti,ab #5 (pain near/3 (management or prevent* or relief or treat*)):ti,ab #6 #4 or #5 #7 ((rat* or measurement or scale* or score* or screen* or questionnaire* or test* or assessment* or VAS or NRS or VRS) near/5 pain):ti,ab #8 ("double-blind" or "treatment outcome" or "outcome assessment"):ti,ab #9 #7 or #8 #3 AND #6 AND #9  Publication Year from 1990 to 2016 | |

**5. PubMed (complement)**

| Date of Search: 2016.03.07  Number of hits: 156  Comments: | Field tags:   - [tiab] = title & abstract |
| --- | --- |
| ("labor pain"[tiab] or "labour pain"[tiab] or labor[tiab] or labour[tiab] or childbirth[tiab] or parturition[tiab] or "obstetric* delivery"[tiab] or "vaginal delivery"[tiab])  AND (analgesi*[tiab] or anesthetic* [tiab] or acupress*[tiab] or acupuncture[tiab] or “nerve block”[tiab] or bath*[tiab] or breathing[tiab] or electroacupuncture[tiab] or homeopathy[tiab] or hydrotherapy[tiab] or hypnosis[tiab] or immersion[tiab] or massage[tiab] or phytotherapy[tiab] or relaxation[tiab] or TENS[tiab] or non-pharmacologic*[tiab] or “nerve stimulation”[tiab] or "sterile water"[tiab] or “pain management”[tiab] or “pain prevention”[tiab] or ”Pain relief”[tiab] or “pain treatment”[tiab]) AND  (rate[tiab] or rating*[tiab] or “pain measurement”[tiab] or scale*[tiab] or score*[tiab] or screen*[tiab] or questionnaire*[tiab] or tests[tiab] or “pain assessment”[tiab] or VAS[tiab] or NRS[tiab] or VRS[tiab] or double-blind[tiab] or outcome[tiab])   NOT Medline[sb]  Filters: Publication date from 1990/01/01 to current; English | |

**6. Web of Science (Thomson Reuters)**

| Date of Search: 2016.03.07  Number of hits: 428  Comments: | Field tags:   - TOPIC = title, abstract, keywords |
| --- | --- |
| TOPIC: (((Labor or labour) NEAR/3 pain) OR (labor or labour or childbirth or parturition or ((obstetric* or vagin*) NEAR/3 delivery)))  AND  TOPIC: ((analgesi* or anesthet* or acupress* or acupuncture or nerve block or bath* or breathing or electroacupuncture or homeopathy or hydrotherapy or hypnosis or immersion or massage or phytotherapy or relaxation or TENS or non-pharmacologic* or “nerve stimulation” or “sterile water”) OR (pain NEAR/3 (management or prevent* or relief or treat*)))  AND  TOPIC: (((rat* or measurement or scale* or score* or screen* or questionnaire* or test* or assessment* or VAS or NRS or VRS) NEAR5 pain) OR (“double-blind” or “treatment outcome” or “outcome assessment”))  Timespan: 1990-2016. Limited to languages: English | |
