# Supplementary material for: Labour Q1 pain – poorly analysed and reported: a systematic review
Source: BMC Pregnancy Childbirth. 2018 Dec 7;18:483. doi: 10.1186/s12884-018-2089-2 (PMC6286546; doi:10.1186/s12884-018-2089-2)
Supplement: Supplementary file 2 — Table S1. Included Journals. (DOCX 14 kb) [file 12884_2018_2089_MOESM2_ESM.docx]

**Additional file 2: Table S1.** Included Journals

|  | | Number | Percent (%) |
| --- | --- | --- | --- |
|  | Anesthesia & Analgesia | 17 | 12.8 |
|  | International Journal of Obstetric Anesthesia | 17 | 12.8 |
|  | Anesthesiology | 16 | 12.0 |
|  | Canadian Journal of Anaesthesia | 9 | 6.8 |
|  | Journal of Clinical Anesthesia | 7 | 5.3 |
|  | Anaesthesia | 5 | 3.8 |
|  | Acta Obstetricia et Gynecologica Scandinavica | 3 | 2.3 |
|  | Archives of Gynecology and Obstetrics | 3 | 2.3 |
|  | British Journal of Anaesthesia | 3 | 2.3 |
|  | Middle East Journal of Anesthesiology | 3 | 2.3 |
|  | Acta Anaesthesiologica Scandinavica | 2 | 1.5 |
|  | British Journal of Anaesthesia | 2 | 1.5 |
|  | International Journal of Gynaecology & Obstetrics | 2 | 1.5 |
|  | Iranian Journal of Nursing and Midwifery Research | 2 | 1.5 |
|  | Journal of Midwifery & Women's Health | 2 | 1.5 |
|  | Regional Anesthesia & Pain Medicine | 2 | 1.5 |
|  | Anaesth Intensive Care | 1 | .8 |
|  | Birth: Issues in Perinatal Care | 1 | .8 |
|  | Bjog-an International Journal of Obstetrics and Gynaecology | 1 | .8 |
|  | Chin J Integr Med | 1 | .8 |
|  | Drug Research | 1 | .8 |
|  | Egyptian Journal of Anaesthesia | 1 | .8 |
|  | European Journal of Anaesthesiology | 1 | .8 |
|  | European Journal of Pain | 1 | .8 |
|  | Gineco Ro | 1 | .8 |
|  | Global Journal of Health Science | 1 | .8 |
|  | Health Science Journal | 1 | .8 |
|  | Indian Journal of Anaesthesia | 1 | .8 |
|  | Int J Ther Massage Bodywork | 1 | .8 |
|  | International Medical Journal | 1 | .8 |
|  | Internet Journal of Anesthesiology | 1 | .8 |
|  | Journal of Anaesthesiology. Clinical Pharmacology | 1 | .8 |
|  | Journal of Caring Sciences | 1 | .8 |
|  | Journal of Nursing Research | 1 | .8 |
|  | Journal of Obstetrics and Gynaecology Research | 1 | .8 |
|  | Journal of Physiotherapy | 1 | .8 |
|  | Journal of Psychosomatic Obstetrics and Gynecology | 1 | .8 |
|  | Journal of the American Osteopathic Association | 1 | .8 |
|  | Journal of the Medical Association of Thailand | 1 | .8 |
|  | Korean Journal of Anesthesiology | 1 | .8 |
|  | Makedonska Akademija na Naukite i Umetnostite Oddelenie Za Bioloshki i Meditsinski Nauki Prilozi | 1 | .8 |
|  | Medical Journal Armed Forces India | 1 | .8 |
|  | Medical Science Monitor | 1 | .8 |
|  | Medicine | 1 | .8 |
|  | Midwifery | 1 | .8 |
|  | Minerva Anestesiologica | 1 | .8 |
|  | Nurs Res | 1 | .8 |
|  | Nursing Journal of India | 1 | .8 |
|  | Pain Research & Management | 1 | .8 |
|  | Regional anesthesia | 1 | .8 |
|  | Rev Bras Anestesiol | 1 | .8 |
|  | Saudi J Anaesth | 1 | .8 |
|  | Shiraz E Medical Journal | 1 | .8 |
|  | Upsala Journal of Medical Sciences | 1 | .8 |
|  | Total | 133 | 100.0 |
