# Supplementary material for: Labour Q1 pain – poorly analysed and reported: a systematic review
Source: BMC Pregnancy Childbirth. 2018 Dec 7;18:483. doi: 10.1186/s12884-018-2089-2 (PMC6286546; doi:10.1186/s12884-018-2089-2)
Supplement: Supplementary file 3 — Appendix S2. Data extraction sheet used to collect data. (DOCX 32 kb) [file 12884_2018_2089_MOESM3_ESM.docx]

**Additional file 3: Appendix S2.** Data extraction sheet used to collect data.

| **Paper information** | | | |
| --- | --- | --- | --- |
| 1. **Article ID**   **(EndNote id)** |  | 4) Does the paper need to be discussed further?   - No - Yes   5) If Yes, specify: | |
| 1. **Name of author (last name)** |  |  |  |
| 1. **Reviewer** | - Linda Vixner - Hans Järnbert-Pettersson |  |  |
| **INCLUSION CRITERIAS / Measures of labor pain** | | | |
| 1. **Paper published** | Year,___________ | |  |
| 1. **Is labor pain compared between two or more groups?** | - No (inclusion not fulfilled) - Yes,   - Number of groups compared___________________ - Not clearly described | | **IF YES, CONTINUE WITH 8.** Focus on the continous outcome of labour pain.  **IF NO or NOT CLEAR,** End examination! |
| 1. **Time points – is labor pain measured at two or more time points in each of the groups?**   (i.e. would it be possible to compare changes between the time point within each group) | - No (inclusion not fulfilled) - Yes,   - Number of times that pain is measured at least___________ - Not clearly described | | **IF YES, CONTINUE WITH 9.** Focus on the continous outcome of labour pain.  **IF NO or NOT CLEAR,** End examination! |
| 1. **Is labor pain analysed as a continuous outcome or presented somewhere in the article as mean?** | - Yes, (analysed as continuous or presented as mean) - No,   - Only median presented analysed   - only analyzed as Categorized   - presented as percentage above or below cutpoint   - Time to event (e.g. pain relief (defined as cutpoint).   - Time to extra dose   - Duration of pain relief   - Number of extra doses of pain relief   - Other,________________________   - Not measured (e.g. duration of labour)   (Exlusioncriteria)  ______________________________   - Not clear | | **IF YES, CONTINUE WITH 10.** Focus on the continous outcome of labour pain.  **IF NO or NOT CLEAR,** End examination! |
| 1. **Are there equally spaced time intervals?** (e.g. every 30 minutes after first treatment given). | - No, Specify interval__________________ - Yes, Specify interval__________________ - Not clearly described | | Report interval as e.g.  (0, 10, 45, 60 min,…)  (0, 15, 30,…) |

**METHODS**

| - **MEASURES OF LABOUR PAIN** | | | | |
| --- | --- | --- | --- | --- |
| 1. **Study design**   C3a  S4 | - Experiment   - Randomized   - Not randomized - Observational   - Cohort   - Cross Sectional   - Other,__________________ - Not clearly defined | |  | |
| 1. **Outcome - is continuous labour pain defined as primary or secondary outcome measures?**   C6a | - Primary outcome/measure - Secondary outcome/measure - measured but not explicitly mentioned as primary/secondary (including main outcomes) - Not clearly described,_________________ | |  | |
| 1. **Is the variable that measure labor pain clearly defined, including used scale?**   S7,  S8 | - No - Yes   - VAS (Visual analogue scale/score)   - NRS (Numeric rating scale, e.g. 1-10)   - MPQ     - McGill, Pain Questionnaire     - McGill, Short form   - VRS (Verbal rating scale)   - PPI (Present pain Intensity)   - Other,________________ | | - If Yes, scale - Min_____ - MinText__________ - Max_____ - MaxText__________ - Number of   Steps on scale_____   - Not clearly defined scale______ | |
| **At what time points was labor pain measured?** | | | | |
| 1. **Was labor pain measured retrospectively or in real time**   C6a, S8 | - Real time - Retrospectively - Not clearly described | |  | |
| - **ANALYSES / COMPARISONS between groups (e.g. placebo vs treatment group)** | | | | |
| 1. Which **statistical methods were used to compare continuous labor pain between groups?**   C12a, C12b  S12a, S12b | - Not clear how comparisons between groups were made, (e.g. assumptions needed) - Chi-Square,___________________ - Fisher,_______________________ - McNemar_______________________ - Mann Whitney U__________________ - Wilcoxon________________________ - T-test   - (not specified dependent/independent)   - T-test (dependent/paired) _____________   - T-test (independent/unpaired)   ________   - ANOVA_________________________ - ANCOVA_________________________ - Repeated measures ANOVA_________ - MANOVA________________________ - Logistic regression_________________ - Linear regression__________________ - Mixed Models_____________________ - Generalized Estimation Equation_______ - Other,___________ | Name instrument if more than one measure of labor pain is used, e.g. VAS, Mcgill.  (inclusion criteria that comparisons are being made, thus all papers should have comparison between groups with respect to labor pain) | | |
| **COMPARISONS within groups (e.g. baselineT0 vs timepoint T3) - Statistical methods** | | | | |
| 1. **Is comparisons made within groups over time?** (i.e. is comparisons made between time points within groups?)   **If yes, how is the comparisons made?**  C12a, C12b  S12a, S12b | - No, __________________ - Yes - Not clear how comparisons are made (assumptions needed) - Chi-Square,____________________ - Fisher,__________________________ - McNemar_______________________ - Mann Whitney U_________________ - Wilcoxon_______________________ - T-test   - not specified dependent/dependent)   - T-test (dependent/paired) ___________   - T-test (independent/Unpaired) - ANOVA_________________________ - ANCOVA________________________ - Repeated measures ANOVA________ - MANOVA_______________________ - Logistic regression________________ - Linear regression_________________ - Mixed Models____________________ - Generalized Estimation Equation_____ - Other,___________ | | | Comment: |
| 1. **Most advanced statistical method used to analyze labor pain for continuous variable?** | - Not clear - End point analysis (t-test, ANOVA, ANCOVA,   MANOVA, Mann whitney)   - rANOVA (r=repeated) - rMANOVA (r=repeated) - Mixed-effect models - GEE - Other,_______________________________ | | |  |

RESULTS

| **Outcome data / Main results – result section** | | |
| --- | --- | --- |
| 1. **Total number of included subjects (women) in the analysis.** | Total:_________________ | Number of included women at the first measurement (baseline). |
| 1. **Is result for continuous labor pain clearly described in each group (effect size)?**   C17A  S16a, S15 | - No, not clearly defined - Yes   - Mean or difference in mean   - Median or difference in median - Other,_________________________ | Presentation in a graph or table is ok, divided by group at specific timepoints.  (e.g. mean pain at time t1 and t2 for each treatment groups). |
|  |  |  |
| **Lost-to-follow up, missing data - result section** | | |
| 1. **Numbers analyzed:**   **Is missing data on labour pain at each time point presented?**  C16,  S14b,S15 | - No   - Only number of included individuals in e.g. tables and figures.i - Yes   - Number of missing data for labor pain at each timepoint is presented   - Number of valid data for labor pain is presented at each time point.   - Number of individuals at each time point, but not clear if valid data for labour pain at each measurement. - Not clearly described,______ | Yes, e.g. if a flow chart is presented that shows how many individuals that was included and how many that was analysed.  Note distinction between lost to follow up and (partial) missing data. |
| 1. **Are normality assumptions tested?** | - No, Not mentioned - Yes,   - Specific test mentioned: e.g. Kolmogorov-Smirnov, Shapiro Wilks   - No specific test mentioned or informal method used(e.g. visual inspection) |  |
| **Discrepance between statistical method section and the presented results** | | |
| 1. **Discrepancies between Statistical method section and the Result section regarding analyzis of labour pain?** | - No, Methods presented in the statistics section is also clearly presented in the results section. - Yes,   - Results and methods used in the result section are not presented in the statistical section.   - Not clear, which methods that have generated e.g. a P-value in the result section. |  |

C=Consort item, S=Strobe item,

For example: C6a correspond to question 6a in the Consort checklist “Completely defined pre-specified primary and secondary outcome measures, including how and when they were assessed”.
